# Myasthenia Gravis With Chronic Kidney Disease: A Diagnostic Challenge

**DOI:** 10.1002/ccr3.72758

**Published:** 2026-05-20

**Authors:** Zahra Mahmood, Amina Mahmud, Aymar Akilimali

**Affiliations:** ^1^ Department of Nephrology Jinnah Postgraduate Medical Centre Karachi Pakistan; ^2^ Dow University of Health Sciences Karachi Pakistan; ^3^ Department of Research Medical Research Circle (MedReC) Goma Democratic Republic of the Congo

**Keywords:** chronic kidney disease, glomerulonephritis, myasthenia gravis, neuromuscular junction disorder

## Abstract

The coexistence of myasthenia gravis (MG) and chronic kidney disease (CKD) presents a diagnostic challenge due to overlapping clinical features. MG is an autoimmune neuromuscular junction disorder, whereas CKD exhibits the gradual loss of kidney function. We report a 65‐year‐old male with long‐standing CKD who developed progressive neuromuscular symptoms, including bilateral ptosis and bulbar involvement. His anti‐acetylcholine receptor antibodies were negative; however, electrophysiological studies demonstrated a significant decremental response (> 10%) on repetitive nerve stimulation, supporting the diagnosis of MG. The patient required hemodialysis for worsening renal function and was treated with pyridostigmine for MG symptoms, resulting in clinical improvement. This case emphasizes the importance of distinguishing MG from CKD‐related symptoms, particularly in seronegative patients, and highlights the need for careful clinical and electrophysiological evaluation.

## Key Clinical Message

This case highlights the challenges of diagnosis and management in complex comorbid conditions. In patients with chronic kidney disease, nonspecific symptoms such as fatigue and weakness may mimic myasthenia gravis. Recognition of neuromuscular junction involvement, even in seronegative cases, is essential to ensure timely management, avoiding misdiagnosis.

**Abbreviations**
CKD: chronic kidney disease
EMG: electromyography
MG: myasthenia gravis
RNS: repetitive nerve stimulation

## 1 Introduction

Chronic kidney disease (CKD) is defined as progressive impairment of renal function, or an eGFR less than 60 mL/min/1.73m^2^, sustained for 3 months or more [1]. Myasthenia gravis (MG) is an autoimmune neuromuscular junction disease characterized by muscle weakness and typical involvement of extraocular, bulbar, and proximal limb muscles.

The coexistence of CKD and MG is uncommon, and overlapping clinical features may complicate diagnosis. We report the case of seronegative MG in a patient with CKD, highlighting diagnostic complexity and the clinical considerations in such presentations when distinguishing neuromuscular junction pathology from uremia‐related symptoms.

## 2 Case Presentation

A 65‐year‐old Pakistani male, with a 15‐year history of hypertension and a 10‐year history of CKD (baseline creatinine of 3.0 mg/dL), presented with bilateral pedal edema, decreased urine output, and exertional shortness of breath for 1 week. Two months before admission, the patient had been diagnosed with MG, based on clinical features and electrophysiological findings.

On examination, the patient was hemodynamically stable with BP 100/60 mmHg, pulse 86 beats per minute, and respiratory rate of 18 breaths per minute. Systemic examination revealed bilateral pedal edema. On auscultation, his chest was bilaterally clear, and heart sounds were audible. His abdomen was soft and nontender. Neurological examination demonstrated bilateral ptosis, nasal speech, and bulbar involvement, with preserved limb strength and no focal neurological deficits.

### 2.1 Investigations and Treatment

Laboratory evaluation showed worsening renal function, with creatinine rising to 8.4 mg/dL and urea to 217 mg/dL (Table 1). Arterial blood gas analysis (ABGs) showed severe metabolic acidosis (pH 7.29, bicarbonate 6.7 mmol/L, and pCO2 14 mmHg), consistent with advanced renal failure.

**TABLE 1:** Complete blood count and renal functions before and after hemodialysis.

Parameter	At admission	After 8 sessions of hemodialysis
Hemoglobin (g/dL)	8.7	9.9
WBCs (cell × 10^9^/L)	11	8.7
Platelets (cell × 10^9^/L)	166	156
Urea (mg/dL)	217	154
Creatinine (mg/dL)	8.4	6.4

Given refractory metabolic acidosis and worsening volume overload, including pulmonary edema, hemodialysis was initiated. Following the first session, clinical improvement in dyspnea was noted; however, shortly afterwards, the patient developed hypotension (50/30 mmHg) and required norepinephrine support, initiated at approximately 0.05 μg/kg/min. Hemodynamic stability improved over subsequent days, and inotropic support was gradually tapered off over 8 days.

**FIGURE 1:**
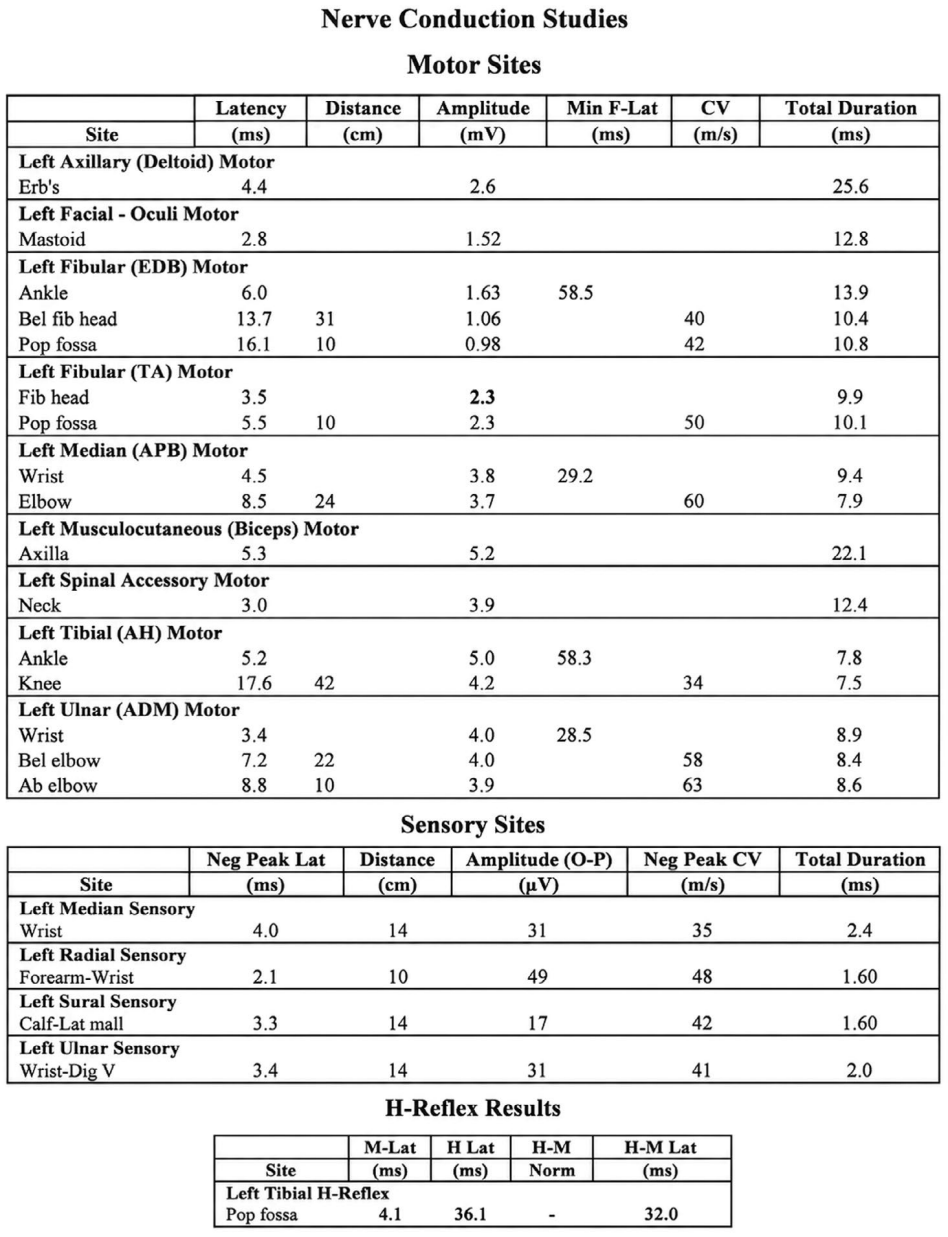
Nerve conduction study (NCS) demonstrates delayed latencies and reduced amplitudes in multiple motor nerves.

**FIGURE 2:**
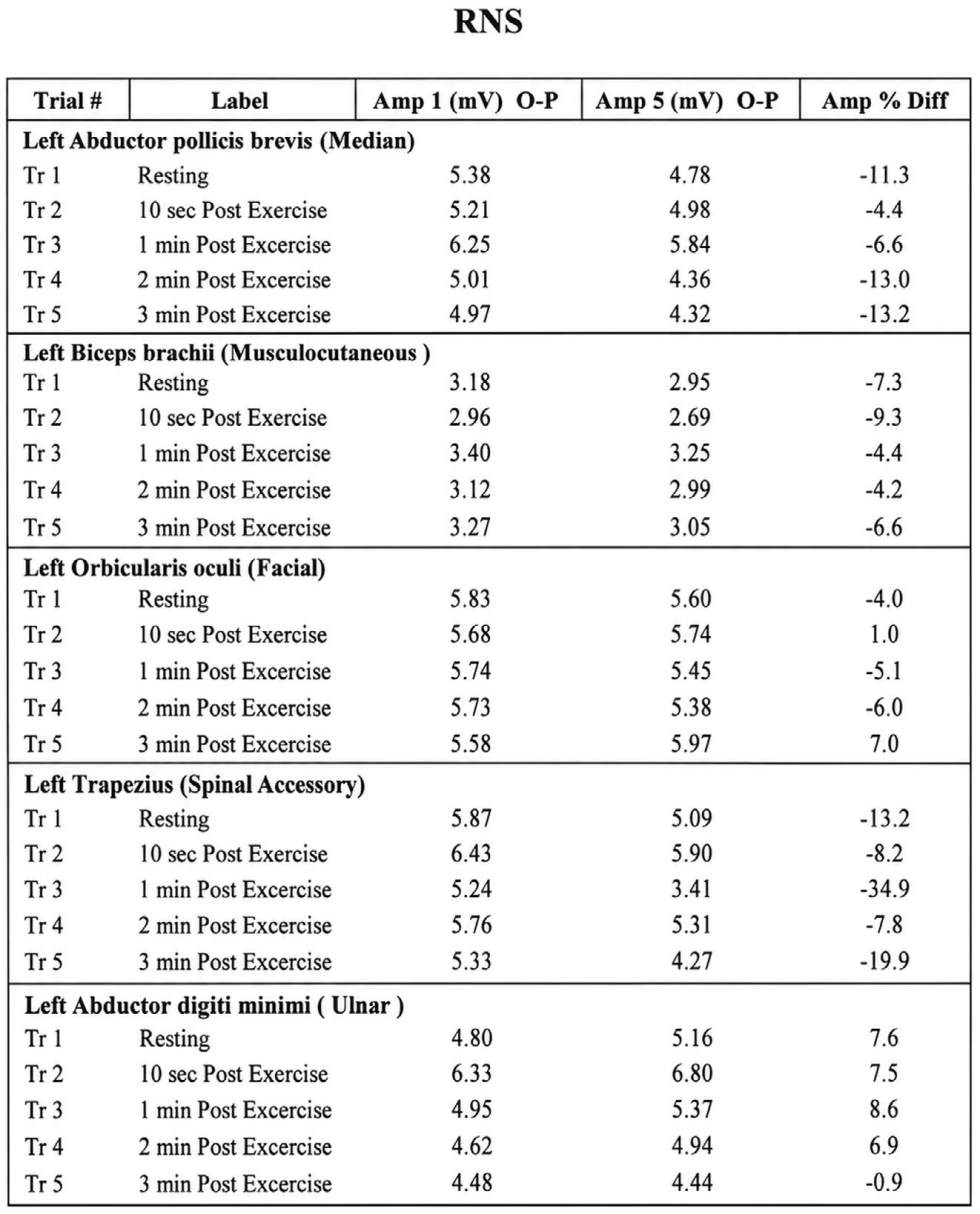
Repetitive nerve stimulation (RNS) demonstrates a significant decremental response, supporting a postsynaptic neuromuscular junction disorder.

**FIGURE 3:**
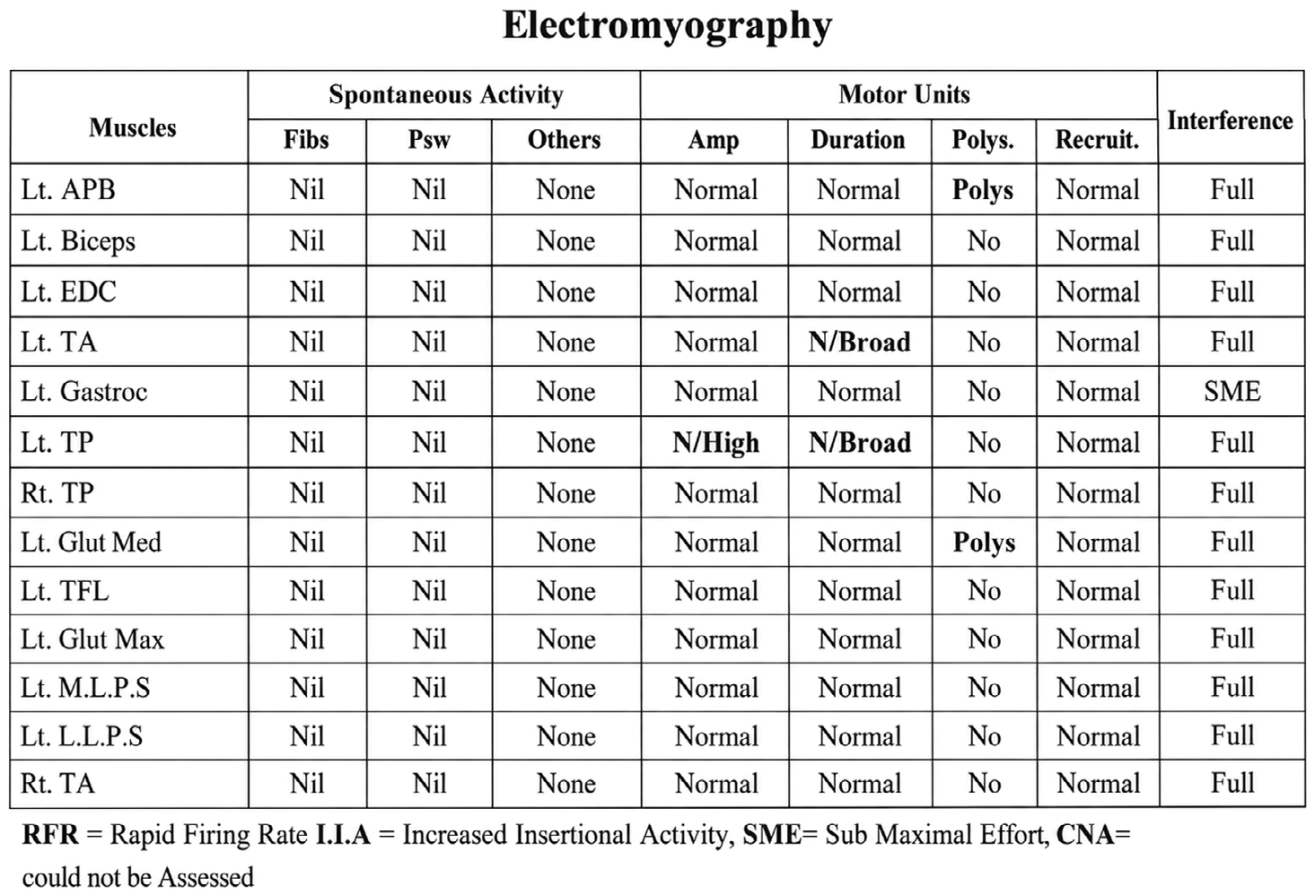
Needle electromyography (EMG) findings show mild chronic neurogenic changes.

**FIGURE 4:**
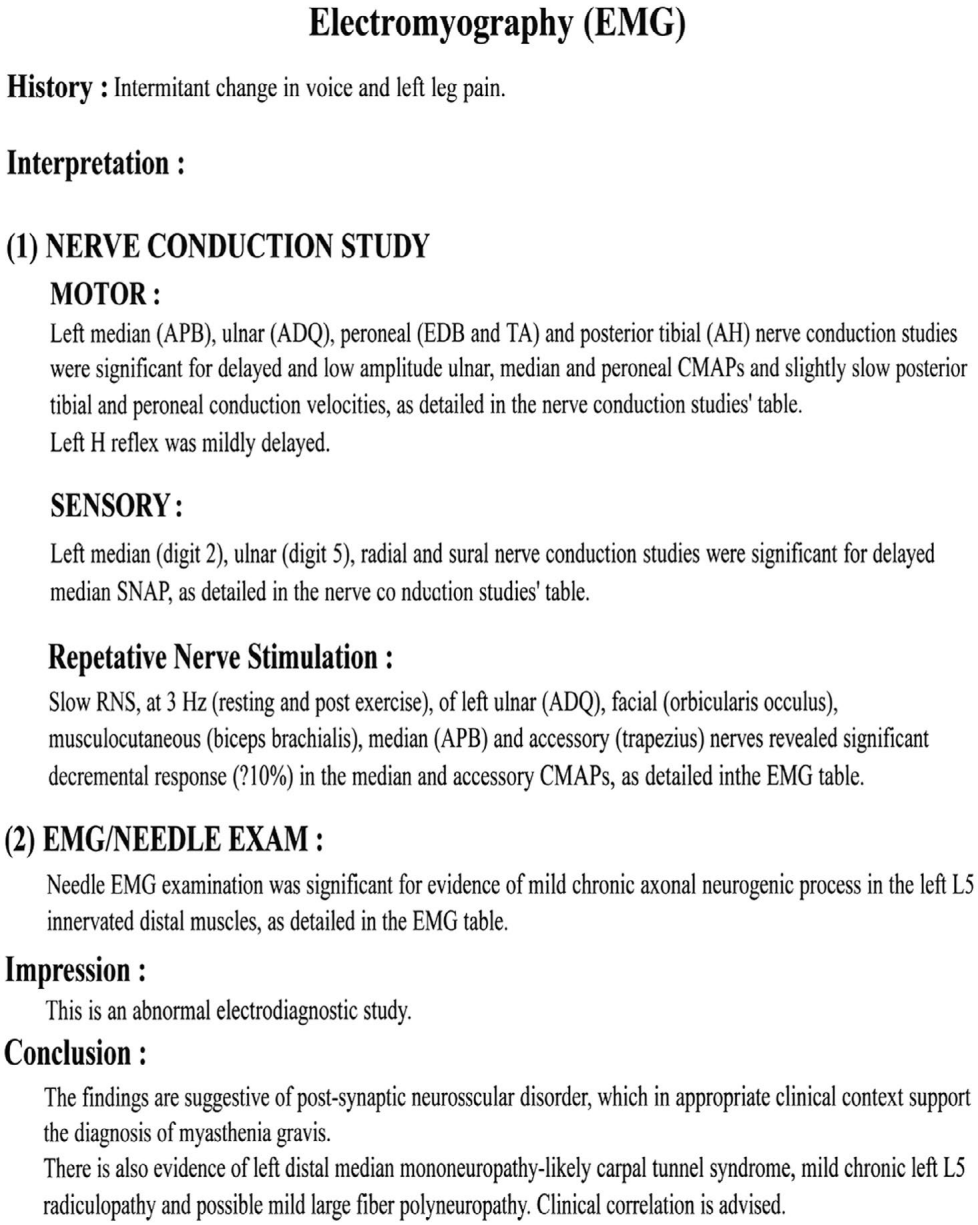
Summary of the electrophysiological study.

Electrophysiological evaluation demonstrated findings consistent with a postsynaptic neuromuscular junction disorder. Nerve conduction studies showed delayed latencies and reduced amplitudes in multiple motor nerves (Figure 1). Repetitive nerve stimulation (RNS), at 3 Hz (resting and postexercise), revealed a significant decremental response exceeding 10% in median and accessory nerves (Figure 2). Needle electromyography (EMG) demonstrated mild chronic neurogenic changes (Figure 3). The report summary is shown in Figure 4.

Anti‐acetylcholine receptor antibodies (Anti AChR‐Ab) were negative (< 0.25 nmol/L), and anti‐MuSK antibody testing was not available in the local hospital setting. The patient was treated with pyridostigmine, with noticeable improvement in ptosis and bulbar symptoms during hospitalization.

Other investigations included ultrasound of the kidneys, ureters, and bladder, which showed bilateral renal parenchymal disease with grade 2 and 3 changes. Echocardiography showed preserved left ventricular systolic function (ejection fraction 60%) with grade 1 diastolic dysfunction.

MRI brain imaging was not performed, as there were no focal neurological deficits suggestive of central pathology. Kidney biopsy was not performed due to the long‐standing nature of CKD and clinical considerations suggesting chronic irreversible disease. Due to patient instability, CT scan for thymoma evaluation was not performed.

### 2.2 Outcome and Follow‐Up

A total of eight hemodialysis sessions were performed. The metabolic parameters stabilized, and uremic symptoms improved significantly (Table 1). Notable improvements in ptosis and bulbar symptoms were observed following the initiation of pyridostigmine, supporting a neuromuscular junction etiology. The patient was discharged in stable condition with plans for outpatient follow‐up, and information regarding the continuation of maintenance dialysis after discharge was not available.

## 3 Discussion

The coexistence of MG and CKD is uncommon. This case highlights the diagnostic challenge of identifying MG in a patient with advanced CKD. Khan et al. suggested that many patients with end‐stage renal disease (ESRD) have signs and symptoms that can be confused with a neuromuscular junction disorder, including easy fatigability, fluctuating weakness, apathy, dry mouth, and blurring of vision [2].

In this patient, although symptoms such as fatigue, weakness, and dyspnea could be attributed to CKD, the presence of bilateral ptosis, bulbar involvement causing slurred and nasal speech, and a significant decremental response on repetitive nerve stimulation strongly supported a diagnosis of MG. These findings, particularly the decrement exceeding 10% observed in testing (Figure 2), are characteristic of a postsynaptic neuromuscular junction disorder and are not explained alone by uremia or metabolic derangements.

The absence of detectable anti‐AChR‐Ab posed an additional diagnostic challenge. However, seronegative MG is a recognized entity, and electrophysiological studies remain central to diagnosis in such cases [3].

Renal involvement in MG has been described in the literature, mainly in association with glomerulonephritis [4] and thymoma [5]. Leading to progressive decline in renal function, minimal change disease, focal segmental glomerulosclerosis, and membranous nephropathy are among the types of glomerulonephritis reported in association with MG [4]. The pathophysiological mechanisms for these associations are unclear. Autoimmune dysregulation, lying at the core of MG and overlapping inflammatory profiles, such as elevated cytokine levels in CKD [6] and MG [7], may be contributing factors. CKD also leads to persistent low‐grade inflammation due to uremic toxins disrupting immune homeostasis [8]. This inflammatory state may worsen MG by amplifying autoimmune responses. Conversely, MG‐related inflammation could exacerbate CKD by promoting vascular endothelial damage and fibrosis.

Literature shares insights into variable clinical courses and management strategies for patients with both MG and advanced renal disease. Kim et al. described a favorable patient response to plasmapheresis [9]. Chen et al. explored the fluctuating symptoms of these patients, linking them to the dialysis schedule and electrolyte management [10].

It is important to interpret clinical improvement following dialysis with caution. While dialysis corrects metabolic abnormalities and may improve nonspecific symptoms, the observed improvement in ptosis and bulbar features after initiating pyridostigmine therapy supports the neuromuscular junction component in this patient's presentation.

This case emphasizes the importance of recognizing MG in patients with CKD, where clinical overlap may lead to misdiagnosis. Careful clinical evaluation, supported by electrophysiological testing, remains important. Further research and exploration are required to decipher any possible pathophysiological association of this coexistence.

## 4 Conclusion

The coexistence of myasthenia gravis and chronic kidney disease presents a diagnostic challenge due to overlapping clinical features. This case emphasizes the importance of careful clinical evaluation and electrophysiological testing, particularly in seronegative cases, with early recognition being essential to guide appropriate management.

## Author Contributions

**Zahra Mahmood:** conceptualization, data curation, project administration, writing – original draft, writing – review and editing. **Amina Mahmud:** writing – original draft, writing – review and editing. **Aymar Akilimali:** validation, writing – review and editing.

## Funding

The authors have nothing to report.

## Consent

Written informed consent was obtained from the patient's legal representative to publish the report as per the journal's policy.

## Conflicts of Interest

The authors declare no conflicts of interest.

## Data Availability

Data sharing not applicable, as no datasets were generated or analyzed during the current study.
